# Supplementary material for: Compressive Garments in Individuals with Autism and Severe Proprioceptive Dysfunction: A Retrospective Exploratory Case Series
Source: Children (Basel). 2020 Jul 13;7(7):77. doi: 10.3390/children7070077 (PMC7401870; doi:10.3390/children7070077)
Supplement: Supplementary file 1 [file children-07-00077-s001.pdf]

SUPPLEMENT MATERIAL

Impact of somatosensory orthoses on behavior and postural control in individuals with autism and severe proprioceptive dysfunction: a retrospective case series

Guinchat et al.

Table S1: Systematic search of Ehlers-Danlos Syndrome and retained diagnosis based on the criteria reported by *Malfait et al., 2017*

Hypermobile EDS (hEDS) or Hypermobility Spectrum Disorder HSD criteria

	Generalized joint hypermobility	Soft skin	Hyperextensive skin	Stretch marks	Piezogenic papules	Multiple abdominal hernias	Atrophic scars	Prolapse	Dental crowding, high, arched palate	Arachnodactyly	Arm span-to-height ratio ≥1.05	Mitral valve prolapse	Aortic root dilation	Family history	Muscular pain, joint dislocation	Differential diagnosis excluded	**hEDS**	**HSD**
P1	1	1	0	0	0	0	0	0	0	0	1	0	0	0	?	1	N	**Y**
P2	1	0	1	1	0	0	0	0	1	1	1	0	0	0	1	1	**Y**	N
P3	1	0	1	1	0	0	1	0	1	1	1	0	0	1	?	1	**Y**	N
P4	1	1	1	0	0	0	0	0	0	1	1	0	0	0	1	1	N	**Y**
P5	1	1	1	0	0	0	0	0	1	1	1	0	0	1	0	1	**Y**	N
P6	1	1	1	0	0	0	0	0	0	0	0	1	0	0	1	1	N	**Y**
P7	1	0	1	0	0	0	1	0	1	1	1	0	0	0	1	1	**Y**	N
P8	0	0	0	1	0	0	0	0	0	0	0	0	0	0	?	0	N	N
P9	0	1	0	1	0	0	0	0	0	0	0	0	0	0	0	0	N	N
P10	0	0	0	0	0	0	0	0	0	0	0	0	0	0	?	0	N	N
P11	0	0	0	0	0	0	0	0	1	1	0	0	0	0	?	0	N	N
P12	0	0	0	0	0	0	0	0	0	0	0	0	0	0	?	0	N	N
P13	0	0	0	0	0	0	0	0	0	0	0	0	0	0	?	0	N	N
P14	0	0	0	0	0	0	0	0	0	0	0	0	0	0	?	0	N	N

0: absent criteria; 1: present criteria; ?: unknown (nonverbal subject or inability to name pain); hEDS: hypermobility Ehlers-Danlos syndrome; HSD: hypermobility spectrum disorder; N: no; Y: yes

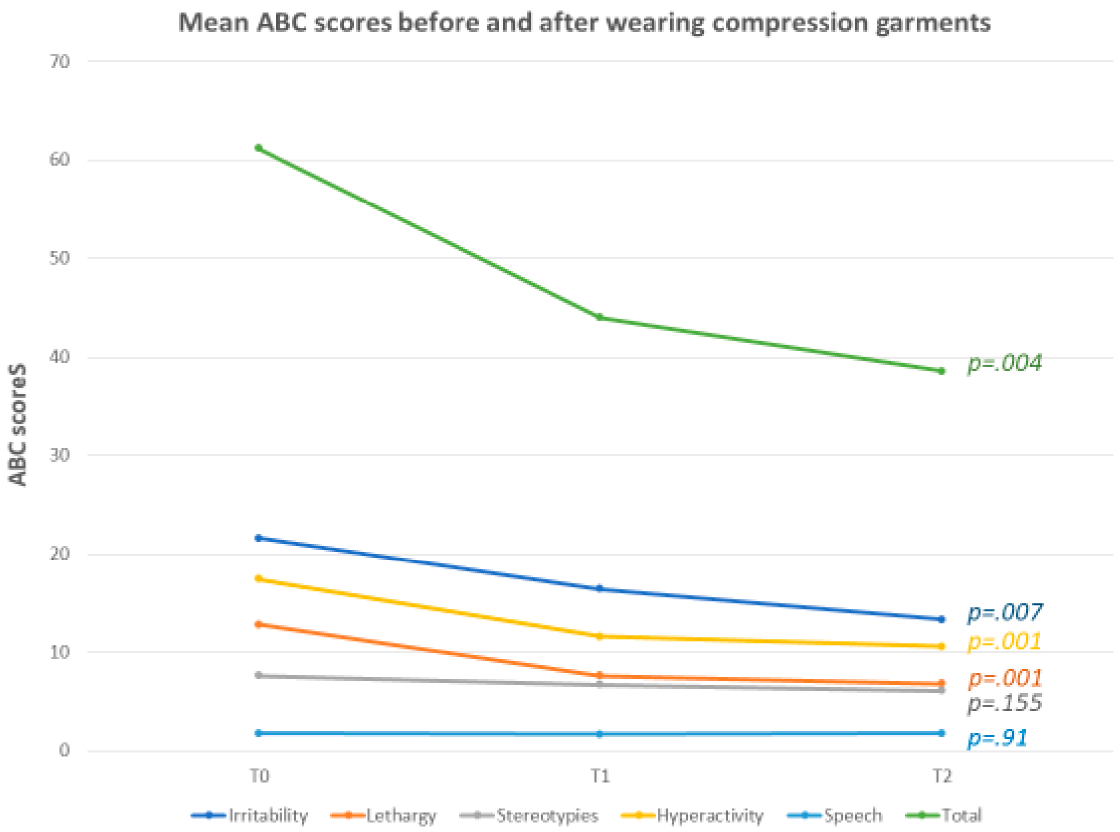

Figure S1. Changes in ABC total scores and subscores between baseline, 2 weeks (T1) and 6 weeks (T2).

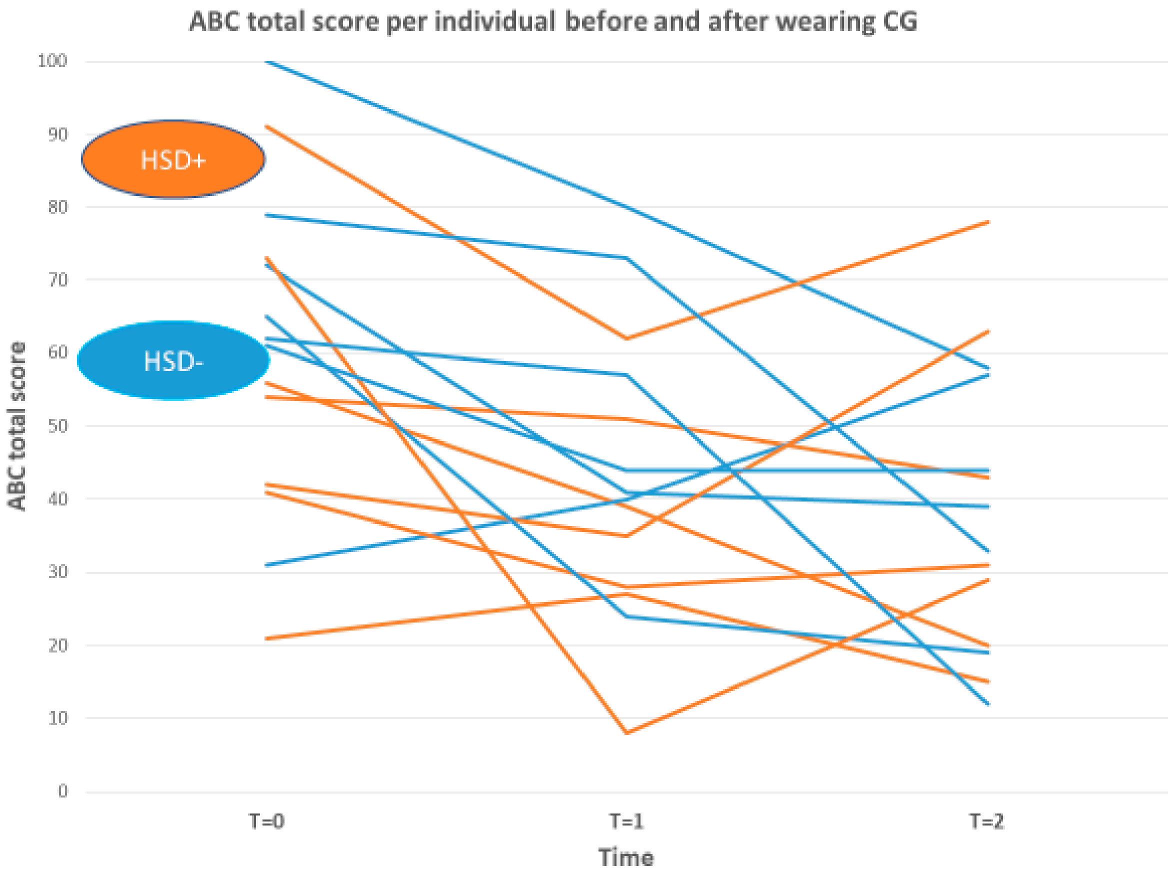

Figure S2. ABC total scores per individual according to the joint hypermobility comorbidity.

HSD: hypermobility spectrum disorder
